# Supplementary material for: Exploring the Information Sources Consulted by Doctors at the Point of Care in Four Selected South African Referral Hospitals
Source: Healthcare (Basel). 2023 Dec 19;12(1):8. doi: 10.3390/healthcare12010008 (PMC10778943; doi:10.3390/healthcare12010008)
Supplement: Supplementary file 1 [file healthcare-12-00008-s001.zip › healthcare-2747990-supplementary.pdf]

APPENDIX A: QUESTIONNAIRE

QUESTIONNAIRE

This study seeks to explore the information behaviour of medical doctors and professional nurses, their tasks, information needs, channels, and sources of information, attitude towards the use of evidence for patient care, and factors that hinder seeking of information by medical doctors and professional nurses. The objectives of the study are as follows:

1. To determine the tasks performed by doctors and nurses in tertiary and central hospitals.
2. To determine the information needs of tertiary and central hospital-based doctors and nurses.
3. To determine the sources of information and communication channels preferred by tertiary and central hospital-based doctors and nurses.
4. To determine the attitude of tertiary and central hospital-based doctors and nurses towards the use of evidence for patient care.
5. To determine the intervening variables that may hinder the seeking of information by doctors and nurses in tertiary and central hospitals.

With your permission, I would like 30 minutes of your time to complete the following questions.

1. **Date of administration (e.g., 10 May 2022):**

____

2. **Demographic Profile**

**2.1. Sex**	**1. Female**	
	**2. Male**	

2. **Date of birth (e.g. 10 August 1935):**

____

**4. Profession**	
1. Medical Doctor	
2. Professional Nurse	
3. Enrolled Nurse	
4. Enrolled Nursing Assistant	

5. **Name of Hospital**

**Nelson Mandela Academic Hospital**	**1**	**Witbank Hospital**	**2**
**Pietersburg Hospital**	**3**	**Robert Mangaliso Sobukwe Hospital**	**4**

6. In which section of the hospital do you work? (e.g., Maternity/Labour ward/Antenal)

____

7. What specific tasks are you performing in your hospital? Please indicate how often you perform those tasks

Tasks	Seldom (1)	Often (2)	Always (3)
1. See patients in outpatients during the day			
2. See patients in casualty during the day			
3. See patients in casualty after hours			
4. Perform ward rounds to see patients admitted in the wards			
5. I work in the theatre			
6. Perform minor procedures			
7. Request and interpret blood investigations to diagnose patient illness			

8. Request and interpret x-ray investigations to diagnose patient illnesses			
9. Prescribe treatment for sick patients			
10. Educate patients about their illness			
11. Give treatment to patients			
12. Review progress of patients on treatment			
13. Teach health workers and health sciences students in the hospital			
14. Review mortality statistics			
15. Conduct folder reviews			
16. Review complaints made by patients			

8. Where do you look to get information in order to meet your needs? Please indicate how often you use that specific source.

	**Seldom (1)**	**Often (2)**	**Always (3)**
1. I talk to colleagues			
2. I consult doctors			
3. I talk to people outside of work			
4. I read newspapers			
5. I use computers at work to access the internet			
6. I use reference books kept in the hospital			
7. I use protocols/guidelines kept in the ward or pocketbook			
8. I consult hospital policy manuals			
9. I use library books			

10. I attend seminars run in the hospital			
11. I attend training workshops organised by non-governmental organisations			
12. I attend training workshops organised by provincial office			
13. I attend training workshops organised by district office			

9. For what reasons do you seek information? Please indicate how often you seek information for a specific reason

**Reasons for seeking information**	**Seldom (1)**	**Often (2)**	**Always (3)**
1. For patient care			
2. For personal use			
3. For CPD (continued professional development)			
4. General awareness			
5. Research			
6. Teaching			
7. Other reasons (Please specify)			

10. What informs your choice of information source? Please tick appropriate box and level of importance

Factors	Somewhat important (1)	Important (2)	Very important (3)
1. Accessibility of the information source			
2. Format of the information source			
3. Cost of the information source			
4. Familiarity with the information source/awareness of the information source			
5. Trustworthiness of the information source			

11. To what extent do you agree with the following statements about your attitude toward evidence-based practices? Please indicate whether you agree or disagree by ticking 1= strongly disagree; 2= disagree; 3= not sure; 4= agree; 5= strongly agree

	**1**	**2**	**3**	**4**	**5**
1. I feel confident that I can perform evidence-based practice					
2. I believe that evidence-based practice leads to improved patient outcomes					
3. I am motivated to adopt evidence-based practice					
4. I make time to keep myself up to date with evidence-based practices					
5. It is easy to apply evidence-based treatment in my day-to-day practice					

12. What factors hinder you in seeking information? Please tick appropriate box and level of importance

Factors	Somewhat important (1)	Important (2)	Very import (3)
1. Time taken to access information			
2. Cost of accessing information			
3. Lack of online access			
4. Lack of skill to search the online resources			
5. Slow internet			
6. Lack of physical library			
7. No print material			
8. Print material not relevant			
9. Do not know how to formulate search questions			

10. Lack of awareness about information sources			
11. Other (Please specify)			

13. How would you rate yourself in the following areas of information use? Please indicate whether you agree or disagree by ticking 1= strongly disagree; 2= disagree; 3= not sure; 4= agree; 5= strongly agree

	**1**	**2**	**3**	**4**	**5**
1. I am aware of all information sources that are available in my hospital					
2. I use all information sources that are available in my hospital					
3. I do not care whether there are information sources provided in my hospital					
4. I always consult information sources provided in my hospital					
5. I consult information sources for clinical decisions					
6. I consult print material (books, journals) from the library					
7. I use computer at work to access online information					
8. I use my book collection					

9. I understand the importance of information at work					
10. I consult information sources for professional development					
11. I consult information sources for teaching					
12. I only consult information sources for personal purposes					
13. Access to information improves quality of patient care					
14. Consulting information sources assist me to diagnose my patients					
15. Consulting information sources assist me in treating my patients					
16. Consulting information sources assist me in the prognosis of my patients					

APPENDIX A: INFORMATION SHEET

Title: Factors influencing the information behaviour of doctors and nurses in South Africa's Eastern Cape, Mpumalanga, Limpopo and Northern Cape provinces: A survey study protocol

Dear sir or madam

The Knowledge Management for Public Health Research Unit, a syndicate of the University of the Witwatersrand, kindly requests you to participate in this study. The completion of the questionnaire will be through your own voluntary measures therefore it will serve as consent to participate. Below is more detailed information about the study.

Purpose of the research: The purpose of this study is to investigate the information behaviour of doctors and nurses in your hospital. It is envisaged that the study will contribute towards providing rich knowledge about the Information behaviour of doctors and nurses in South African tertiary hospitals.

Procedure to be followed: Data will be collected in the form of survey questionnaires. Data collection will commence after we have obtained ethical clearance from the Walter Sisulu University Human Research Ethics Committee. Considering the COVID-19 pandemic, all COVID-19 related regulations will be followed, and the questionnaires will be conducted using online platform.

Discomforts/risks: The risks in this study are minimal (i.e., no greater than those ordinarily encountered in daily life). There are no foreseeable discomforts or dangers to you.

Incentives/benefits for participation: There are no direct benefits to you. The results of this study, however, will increase our knowledge on the Information behaviour of doctors and nurses in your hospital. There will be no cost to you for participating in this study. In addition, you will not be paid, or any other incentives given for your participation.

Time duration of participation: Completing the questionnaire will take 30 minutes of your time. Please note by completing and submitting this online survey means consenting to participate in this study.

Statement of confidentiality: All records are kept confidential and will be available only to professional researchers and staff. If the results of this study are published, the data will be presented in group form and individual or personal identifiers of participants will not be used.

Voluntary participation: Your participation is voluntary, and you are free to withdraw at any time if you wish to do so.

Termination of participation: If at any point you wish to terminate the session, we will do so.

Outputs: If you would like to be informed of the final research findings, please contact any one of the principal investigators (contact details at the end of this information sheet). Findings will be disseminated widely to all stakeholders, annual partner meetings, and peer review journals. If you have any concerns or complaints regarding the ethical procedures of this study, you are welcome to contact the Walter Sisulu University Human Research Ethics Committee (Medical), telephone +27(0) 47 502 2111, email nhangamso@gmail.com/ zdlamini@rtc.wsu.ac.za

Principal investigator: DR ; NOMBULELO CHITHA; Cell 072 218 9258; Email-nchitha@witshealth.co.za

Co-Investigator: DR WEZILE CHITHA; Cell 071 490 8729; Email-wchitha@witshealth.co.za
